# Supplementary material for: Personalized Diet in Obesity: A Quasi-Experimental Study on Fat Mass and Fat-Free Mass Changes
Source: Healthcare (Basel). 2021 Aug 25;9(9):1101. doi: 10.3390/healthcare9091101 (PMC8469514; doi:10.3390/healthcare9091101)
Supplement: Supplementary file 1 [file healthcare-09-01101-s001.zip › healthcare-1309362-supplementary.pdf]

Personalized diet in obesity: a quasi-experimental study on fat mass and fat free mass changes.

Manuel Reig García-Galbis, Diego I. Gallardo, Rosa María Martínez-Espinosa, María José Soto-Méndez.

Table S1. Learning process for adaptation to dietary intervention (adapted from Reig 2015) [37].

**Pre-Initiation period**	
This methodology is based on a dynamic learning process in which it is advisable to schedule several consultations ≥ 2 per month (30 minutes/ consultation approx.). The methodology differentiates three periods: initiation, improvement, and maintenance [37]. The length of each period depends on the magnitude of changes in body weight and fat (changes that will correct metabolic alterations) [8,13,37]	
**Procedures developed at the dietician office**	**Tools for monitoring the intervention**
First consultation [37] (p. 2023)<br>The following procedures were performed:<br>i. To explain the treatment.<br>ii. To measure weight, body fat, blood pressure, biochemical markers and to analyse the results.<br>iii. To recommend the record of relevant data from dietary survey.	Nutritional history [38] (pp. 131-34)<br>Dietary survey [38,39] (p. 137).
**Initiation period** [37] (p. 2023)	
The length of this period depends on the degree of excess weight of:<br>i. Overweight (equivalent to high fat level): the goal is to achieve a change in weight and body fat that helps to reach the healthy level of fat and BMI.<br>ii. Obesity (equivalent to the highest level of fat): the goal is to achieve 5% change in weight and body fat.	
**Procedures developed at the dietician office** [37] (p. 2023)	**Tools for monitoring the intervention**
Second consultation and initiation of the intervention<br>The following procedures were performed:<br>i. To measure weight, body fat, and blood pressure.	Dietary survey [38,39] (p. 137).<br>Personalized diet [38] (pp. 145-147)<br>Recommendations for the initiation of the method [38] (pp. 148-150)

ii. To make agreements with subjects on the objectives (realistic and achievable) to be achieved in short periods (1-2 weeks).	
Until the end of the period of initiation<br>The following procedures were performed:<br>i. To measure weight, body fat, and blood pressure.<br>ii. To monitor the achievement of short-term (1-2 weeks) and medium-term (1-3 months) objectives.<br>iii. To analyse if learning towards healthier eating habits occurs.	Dietary survey [38,39] (p. 137).<br>Personalized diet [38] (pp. 145-147)
**Improvement period** [37] (p. 2023)<br>The length of this period depends on the degree of excess weight of:<br>i. Overweight: the goal is to achieve a 5% change in weight and body fat from the beginning of the intervention.<br>ii. Obesity: the goal is to achieve a change of 10% from the beginning of the intervention (a greater change is recommended as the higher the degree of excess weight and body fat is).<br>In this period, subjects must learn to remove not appropriate behaviours like the following: lack of male interest towards diet; unhealthy diet; lack of time to plan the grocery shopping, preparation, and cooking of healthy food; lack of facilities to store, prepare, and cook healthy food or limited knowledge, skills, and motivation for the incorporation of healthy habits [12]	
**Procedures developed at the dietician office** [37] (p. 2023)	**Tools for monitoring the intervention**
The following procedures were performed:<br>i. To analyse changes in weight, body fat, blood pressure and biochemical markers, if necessary.<br>ii. To observe the ability to communicate the difficulties that have arisen and how faced them.<br>iii. To ask if physical activity was performed and the average number of hours of sleep per day.<br>iv. To monitor the achievement of short-term (1-2 weeks), medium-term (1-3 months) and long-term (1 year) objectives.	Dietary survey [38,39] (p. 137).<br>Personalized diet [38] (pp. 145-147)
**Maintenance period** [37] (p. 2023)<br>The length of this period depends on the degree of excess weight of:<br>i. Overweight or healthy weight: it is recommended to attend the consultation every 3 or 6 months.<br>ii. Obesity: in this case, the following situations could be found:	

a. The degree of obesity has not decreased, but a change of 10% of weight and body fat has been achieved. Consequently, it is recommended to attend the consultation every 1 or 2 months.<br>b. Changes in weight and body fat allow to reach the degree of overweight. Thus, it is recommended to attend the consultation every 3 or 6 months;<br>c. healthy weight and healthy fat are achieved. Consequently, it is recommended to attend the consultation every 6 months.<br><br>Subjects should have learned the essential rules to keep weight and body fat under control over time.	
**Procedures developed at the dietician office** [37] (p. 2023)	**Tools for monitoring the intervention**
The following procedures were carried out:<br>i. To observe changes in weight, body fat, blood pressure and biochemical markers, if necessary.<br>ii. To strengthen the balance between Monday to Friday intakes in a more stringent way compared to weekend intakes (little more energetic meals could be ingested).<br>iii. To reinforce the idea that the physical activity done so far should not be abandoned.<br>iv. To monitor the achievement of medium-term (1-3 months) and long-term (1-2 years) objectives.	Dietary survey [38,39] (p. 137).<br>Personalized diet [38] (pp. 145-147)
